# E2f2 Attenuates Apoptosis of Activated T Lymphocytes and Protects from Immune-Mediated Injury through Repression of Fas and FasL

**DOI:** 10.3390/ijms23010311

**Published:** 2021-12-28

**Authors:** Noor Mustafa, Jone Mitxelena, Arantza Infante, Olatz Zenarruzabeitia, Ainhoa Eriz, Ainhoa Iglesias-Ara, Ana M. Zubiaga

**Affiliations:** 1 Department of Genetics, Physical Anthropology and Animal Physiology, University of the Basque Country, UPV/EHU, 48080 Bilbao, Spain; noor.mustafa01@universitadipavia.it (N.M.); jone.michelena@ehu.eus (J.M.); ainhoa.eriz@ehu.eus (A.E.); 2 Ikerbasque, Basque Foundation for Science, 48009 Bilbao, Spain; 3 Stem Cells and Cell Therapy Laboratory, Biocruces Bizkaia Health Research Institute, 48903 Barakaldo, Spain; arantza.infantemartinez@osakidetza.eus; 4 Immunopathology Group, Biocruces Bizkaia Health Research Institute, 48903 Barakaldo, Spain; olatz.zenarruzabeitiabelaustegui@osakidetza.eus

**Keywords:** E2f, apoptosis, Fas, FasL, T lymphocytes, immune disorder

## Abstract

Targeted disruption of E2f2 in mice causes T-cell hyperactivation and a disproportionate cell cycle entry upon stimulation. However, *E2f2*^−/−^ mice do not develop a lymphoproliferative condition. We report that E2f2 plays a Fas-dependent anti-apoptotic function in vitro and in vivo. TCR-stimulated murine *E2f2*^−/−^ T cells overexpress the proapoptotic genes *Fas* and *FasL* and exhibit enhanced apoptosis, which is prevented by treatment with neutralizing anti-FasL antibodies. p53 pathway is activated in TCR-stimulated *E2f2*^−/−^ lymphocytes, but targeted disruption of p53 in *E2f2*^−/−^ mice does not abrogate Fas/FasL expression or apoptosis, implying a p53-independent apoptotic mechanism. We show that E2f2 is recruited to *Fas* and *FasL* gene promoters to repress their expression. in vivo, *E2f2*^−/−^ mice are prone to develop immune-mediated liver injury owing to an aberrant lymphoid Fas/FasL activation. Taken together, our results suggest that E2f2-dependent inhibition of Fas/FasL pathway may play a direct role in limiting the development of immune-mediated pathologies.

## 1. Introduction

T-cell homeostasis is triggered by signals emanated from the T-cell receptor (TCR) [1]. Upon recognition of a foreign antigen, naïve T cells carrying antigen-specific TCRs grow in size while firing a robust proliferation and differentiation program [2]. This is closely followed by the induction of apoptosis, in order to prevent a hyperactive T-cell population from overreaction once the pathogen has been cleared [3]. Transcriptional regulation has a major role in T-cell homeostasis. We and others have found that individual loss of the transcription factor E2f2, or combined loss of E2f1/E2f2, in mice results in earlier entry of quiescent T cells into S phase following TCR stimulation, and in more extensive proliferation and differentiation [4,5]. Furthermore, *E2f2*^−/−^ mice develop a late-onset disorder with autoimmune features [4]. Some of these effects have been attributed to the transcriptional overexpression of cell cycle genes in knockout animals [6], but the underlying mechanisms remain to be fully elucidated [4].

The control of the potentially limitless expansion of activated T cells is primarily achieved by activation of the death receptor Fas (CD95/Apo-1) [7]. Fas is a cell surface glycoprotein that triggers apoptosis upon binding to its cognate ligand, Fas Ligand (FasL/CD95L), or when experimentally activated by specific agonistic antibodies [8]. Excessive Fas/FasL-mediated apoptosis resulting from abnormal activation and proliferation of T cells has a negative impact in the surrounding tissues, leading to immune-mediated injury. In this regard, tissue-infiltrating T lymphocytes have been implicated in numerous pathologies involving the Fas/FasL pathway, such as Sjogren syndrome, insulitis and diabetes, liver injury, atherogenesis, and graft rejection [9,10,11,12,13]. Thus, a better understanding of Fas/FasL regulation may help mitigate immune-mediated disorders. 

The regulation of *Fas* and *FasL* gene expression plays an important role in the fine-tuning of the Fas/FasL pathway [14,15]. Under stress, transcriptional activation of both *Fas* and *FasL*, and the trafficking of Fas to the cell surface are promoted by p53 in many tumor cells [16,17,18]. Moreover, Fas/FasL expression is increased after ablation of D-type cyclins in murine adult hematopoietic precursor cells through a mechanism involving E2f1 [19]. Little is known, however, about the role of p53 and E2F factors in the regulation of Fas/FasL expression in T lymphocytes, and its impact on T-cell homeostasis. In this work, we have examined the fate of TCR-stimulated *E2f2*^−/−^ T lymphocytes in vitro and in vivo. We show that activated *E2f2*^−/−^ T cells exhibit higher apoptotic rates than their wild-type counterparts through p53-dependent and p53-independent mechanisms involving *Fas* and *FasL* transcriptional upregulation, which sensitize *E2f2*-deficient mice to immune-mediated liver injury. These results point to a novel role for E2f2 as a negative regulator of apoptosis, and could help design new approaches targeting E2F2 for the treatment of immune-mediated human pathologies.

## 2. Results

### 2.1. Increased Apoptosis in TCR-Stimulated E2f2^−/−^ T Lymphocytes

**Figure 1:**
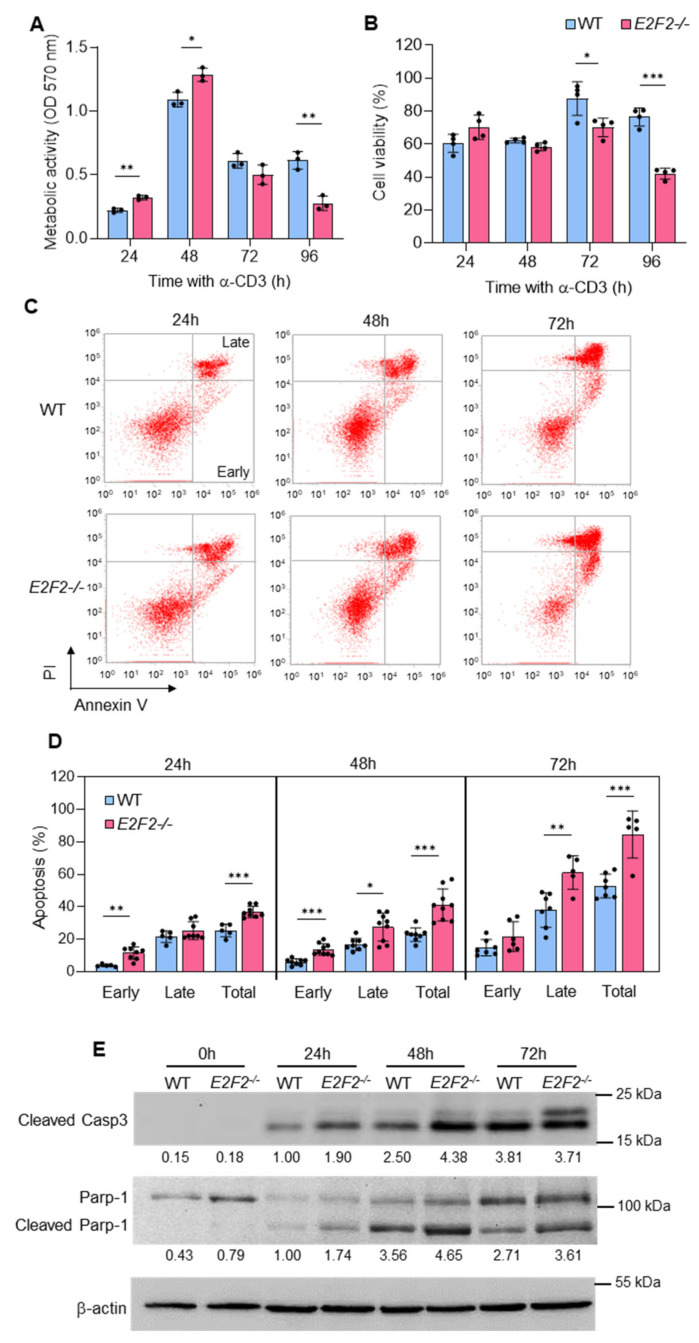
Loss of E2f2 leads to increased apoptosis in TCR-stimulated T lymphocytes: (**A**) Metabolic activity measured by MTT at different times after TCR stimulation with anti-CD3. Results are expressed as optical densities (ODs) at 570 nm from 3 independent experiments. Note that *E2f2*^−/−^ cells exhibit enhanced activity at early time-points, but reduced activity at the final time-point. (**B**) Cell viability assessed by Trypan-blue staining of WT and *E2f2*^−/−^ purified T lymphocytes after stimulation with anti-CD3 for the indicated times. Data (mean ± SD) are indicated as the percentage of viable cells over the number of cells stimulated from 4 independent experiments. (**C**) Apoptotic death in TCR-stimulated T cells assessed by Annexin V-FITC and Propidium Iodide (PI) staining followed by FACS analysis. Different T-cell populations were distinguished as follows: early apoptosis (Annexin V+/PI−); late apoptosis (Annexin V+/PI+); necrosis (Annexin V−/PI+) and viable cells (Annexin V−/PI−). (**D**) Results of early, late, and total apoptosis are expressed as percentage of cells (mean ± SD) from 5 to 9 independent data sets. *** *p* < 0.0001; ** *p* < 0.005, * *p* < 0.05. (**E**) Representative Western blot analysis of Cleaved Caspase 3 and Parp-1 in extracts prepared from freshly purified WT and *E2f2*^−/−^ T cells unstimulated or at different times after stimulation with anti-CD3. Expression of β-actin was used as loading control. Numbers below the bands correspond to the relative densitometric values, expressed as fold-over WT at 24 h. Similar results were obtained in at least 4 independent experiments.

We analyzed the proliferation and viability of purified T lymphocytes derived from the lymph nodes of wild-type (WT) and *E2f2^−/−^* mice at several time points after stimulation with a TCR-activating anti-CD3 antibody. Metabolic activity in stimulated *E2f2*-deficient T lymphocytes was higher than in WT during the first 24–48 h (Figure 1A), consistent with a higher proliferation rate [6], whereas by 96 h of culture, it was substantially lower than in controls. We confirmed that T-cell viability was compromised in *E2f2^−/−^* cells at late time-points (Figure 1B). AnnexinV-PI staining showed an increased rate of apoptotic death in anti-CD3-activated *E2f2^−/−^* cells (Figure 1C,D). This was confirmed by the accumulation of caspase 3 (Casp3) and Parp-1 cleavage fragments, both elevated in *E2f2*-knockouts compared to WT controls (Figure 1E).

We wondered whether the increased apoptosis detected in *E2f2*-null cells could be due to an upregulation of E2f1, a known proapoptotic factor in T lymphocytes [20]. Consistent with our previous findings [4], *E2f1* mRNA and protein levels were increased in *E2f2^−/−^* cells (Supplementary Figure S1A,B). However, E2f1 did not account for the higher apoptotic levels in *E2f2*-deficient T cells, because apoptosis in *E2f1^−/−^/E2f2^−/−^* double knockout (DKO) cells remained unchanged with respect to *E2f2^−/−^* single knockouts (Supplementary Figure S1C). Thus, the increased apoptosis of *E2f2^−/−^* cells can be specifically ascribed to the lack of *E2f2*.

### 2.2. Apoptosis of TCR-Stimulated E2f2^−/−^ T Cells Is Linked to Upregulation of Fas/FasL Gene Expression and Activation of the Fas/FasL Pathway

**Figure 2:**
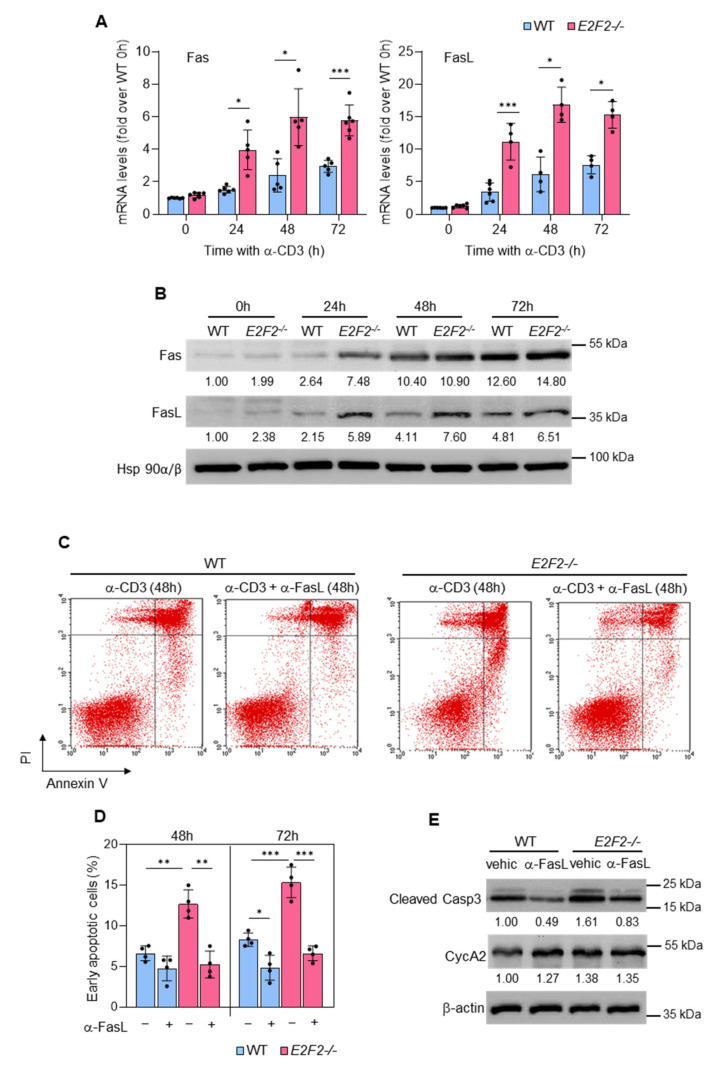
Apoptosis in TCR-stimulated *E2f2*^−/−^ lymphocytes is linked to upregulation of Fas and FasL and activation of the pathway: (**A**) Reverse transcription qPCR analysis of *Fas* and *FasL* expression in freshly purified WT and *E2f2*^−/−^ T cells unstimulated or after stimulation with anti-CD3 during the indicated time. *Eef1a1* was used as normalization control. Results are expressed as fold-over WT at 0 h (mean ± SD) from 3 to 5 independent experiments. *** *p* < 0.0001; * *p* < 0.05. (**B**) Representative Western blot analysis of Fas and Fas L in extracts prepared from freshly purified WT and E2F2^−/−^ T cells unstimulated or after stimulation with anti-CD3 for the indicated time. Expression of Hsp90α/β was used as loading control. Numbers below the bands correspond to the relative densitometric values, expressed as fold-over WT at 0 h. Similar results were obtained in at least 4 independent experiments. (**C**) Representative results of Annexin V-FITC and PI staining followed by FACS analysis performed with WT and *E2f2*^−/−^ T cells after stimulation with anti-CD3 for 48 h and incubation with vehicle or anti-FasL antagonistic antibody for the last 24 h. (**D**) Results of early apoptosis observed under the indicated conditions, expressed as the percentage of cells (mean ± SD) from 4 independent experiments. *** *p* < 0.0001, ** *p* < 0.01. (**E**) Representative Western blot analysis of Cyclin A2 (CycA2) and Cleaved Caspase 3 (Casp3) in extracts prepared from WT and E2f2^−/−^ T cells 48 h after stimulation with anti-CD3 and incubation with anti-FasL antagonistic antibody or vehicle (vehic) for the last 24 h. Numbers below the bands correspond to the relative densitometric values, expressed as fold-over WT vehicle.

We examined whether the differences in apoptosis observed between *E2f2^−/−^* and WT T cells involved the Fas/FasL pathway. Expression of Fas/FasL mRNA and protein increased after TCR activation in WT cells. Remarkably, their levels were significantly higher in *E2f2^−/−^* cells (Figure 2A,B), suggesting a negative regulation of Fas/FasL expression by E2f2. Supporting this possibility, siRNA-mediated silencing of E2F2 in the human cell line HCT116 led to *FAS* mRNA upregulation (Supplementary Figure S2).

Importantly, treatment with neutralizing antibody to FasL reduced apoptosis of anti-CD3-activated WT T cells and completely rescued excess apoptosis of stimulated *E2f2^−/−^* T cells, as shown by Annexin-PI staining (Figure 2C,D) and assessment of cleaved Casp3 (Figure 2E). As a control, we verified that anti-FasL treatment did not affect anti-CD3-mediated activation of the cell cycle, as Cyclin A2 (CycA2) levels remained unchanged upon the incubation (Figure 2E). These results support the notion that TCR-stimulated *E2f2^−/−^* T cells undergo unrestrained apoptosis as a consequence of Fas and FasL upregulation and subsequent activation of the pathway.

### 2.3. p53-Dependent and p53-Independent Mechanisms Mediate Induction of Apoptosis and Fas/FasL Upregulation in E2f2^−/−^ T Lymphocytes

**Figure 3:**
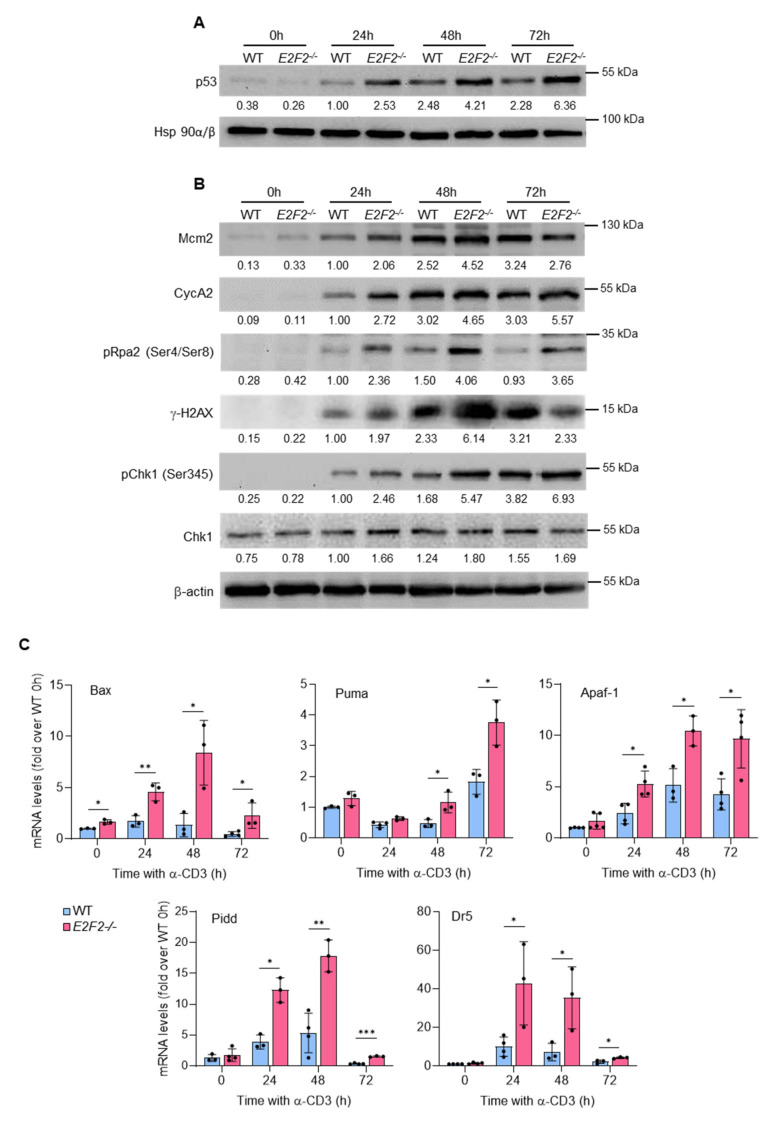
Apoptosis in TCR-stimulated *E2f2*^−/−^ lymphocytes is associated with accumulation of p53 and replication stress: (**A**) Representative Western blot analysis of p53 in extracts prepared from freshly purified WT and *E2f2^−/−^* T cells at several times after stimulation with anti-CD3. Expression of Hsp90α/β was used as loading control. (**B**) Representative Western blot analysis of several replication stress markers. Expression of β-actin was used as loading control. Numbers below the bands correspond to the relative densitometric values, expressed as fold-over WT at 24 h. Similar results were obtained in at least 4 independent experiments. (**C**) Reverse transcription qPCR analysis of p53-target genes in WT and *E2f2*^−/−^ T cells unstimulated or after stimulation with anti-CD3 during the indicated time. *Eef1a1* was used as normalization control. Results are expressed as fold-over WT at 0 h (mean ± SD) from 3 to 5 independent experiments. *** *p* < 0.0001, ** *p* < 0.005, * *p* < 0.05.

After TCR-stimulation, wild-type T cells showed increased p53 protein levels (Figure 3A), expression of DNA replication and cell cycle proteins (Mcm2 and CycA2), as well as an induction of replication stress and DNA damage markers (phospho-Rpa2, γH2AX, and phospho-Chk1) (Figure 3B). Remarkably, TCR-stimulated *E2f2*^−/−^ T cells exhibited significantly higher levels of p53 (Figure 3A), together with a more robust upregulation of DNA replication, cell cycle and DNA damage markers (Figure 3B), suggesting increased levels of genomic instability in *E2f2*-null cells. Interestingly, several p53 transcriptional target genes involved in apoptosis were significantly overexpressed in *E2f2^−/−^* samples (Figure 3C), leading us to consider the possibility that p53-dependent mechanisms could be responsible for the increased apoptosis observed in *E2f2^−/−^* T cells.

**Figure 4:**
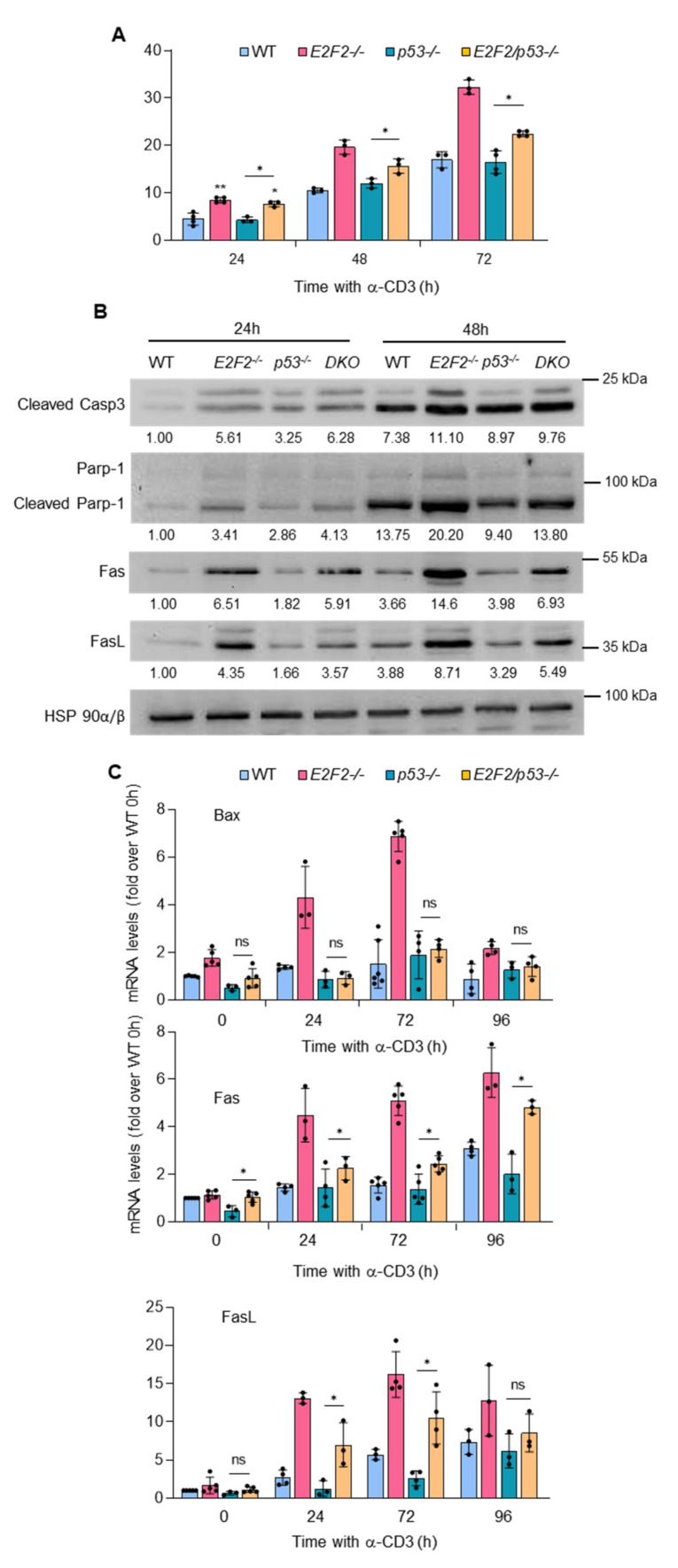
Evidence of p53-independent Fas/FasL upregulation and induction of apoptosis in *E2f2*^−/−^ T lymphocytes: (**A**) Apoptotic death in TCR-stimulated T cells derived from lymph nodes of WT, *E2f2*^−/−^, *p53*^−/−^ and *E2f2^−/−^*/*p53*^−/−^ mice, assessed by Annexin V-FITC and PI staining followed by FACS analysis. Results are expressed as the percentage of cells undergoing early apoptosis (mean ± SD) from 3 independent experiments. (**B**) Representative Western blot analysis of cleaved Caspase 3, Parp-1, Fas, and FasL in extracts prepared from freshly purified WT, *E2f2*^−/−^ *p53*^−/−^ and *E2f2*/*p53*^−/−^ (DKO) T cells unstimulated or after stimulation with anti-CD3 during the indicated time. Expression of β-actin was used as loading control. Numbers below the bands correspond to the relative densitometric values, expressed as fold-over WT at 24 h. Similar results were obtained in 3 experiments. (**C**) Reverse transcription qPCR analysis of *Bax, Fas,* and *FasL* in WT, *E2f2*^−/−^, *p53*^−/−^, and *E2f2*/*p53*^−/−^ purified T cells unstimulated or after stimulation with anti-CD3 during the indicated time. *Eef1a1* was used as normalization control. Results are expressed as fold-over WT at 0 h (mean ± SD) from 5 independent experiments. ** *p* < 0.005, * *p* < 0.05, ns = non-significant.

In order to test this hypothesis, we generated *E2f2*/*p53* DKO mice. Surprisingly, concomitant loss of *p53* only partially restored the percentage of stimulated T cells undergoing apoptosis in *E2f2^−/−^* mice to the levels seen in WT or p53-knockout T cells (Figure 4A). Such a partial rescue was also evident in the levels of apoptotic markers (cleavage of Casp3 and Parp-1) shown by DKO T cells (Figure 4B). mRNA expression of Bax and other well-known p53-specific targets was completely rescued upon loss of p53 (Figure 4C and Supplementary Figure S3). Conversely, Fas and FasL mRNA and protein expression was not restored to WT or p53^−/−^ levels in DKO cells (Figure 4B,C). These results suggest that both p53-dependent and p53-independent mechanisms contribute to the induction of apoptosis and the regulation of Fas/FasL expression in *E2f2*-deficient T cells.

### 2.4. E2f2 Negatively Regulates the Expression of Fas and FasL at the Transcriptional Level

**Figure 5:**
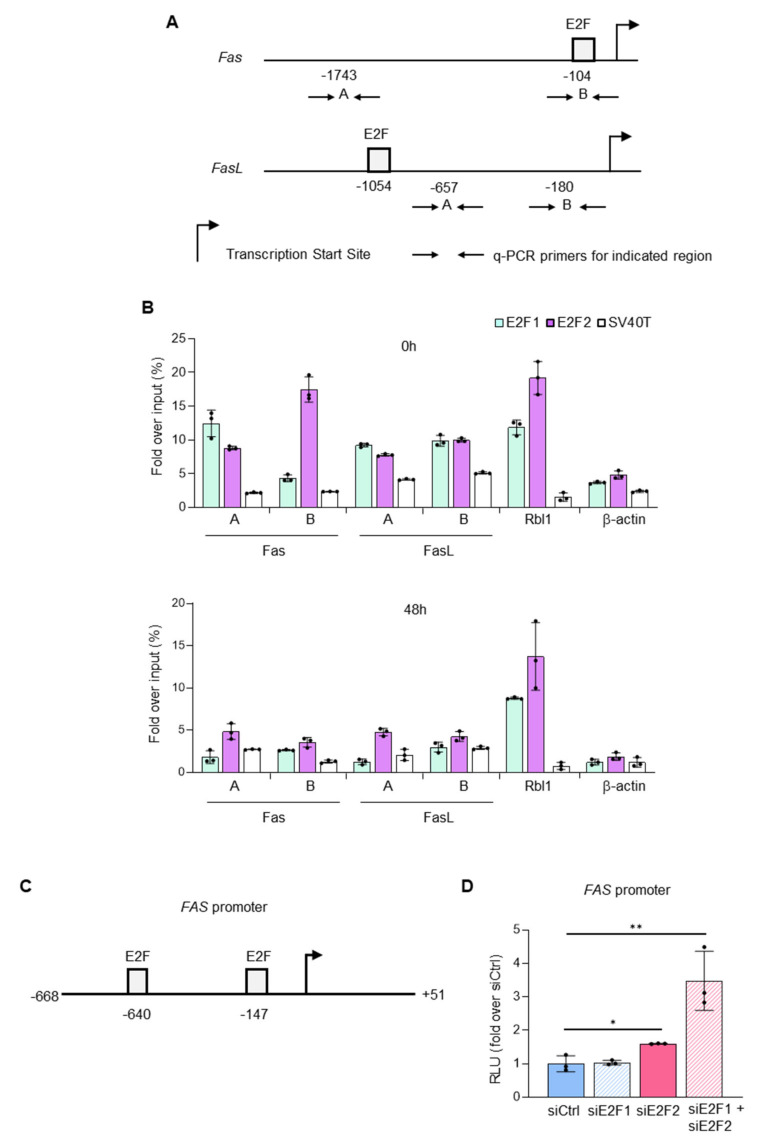
E2F2 regulates expression of Fas and FasL at the transcriptional level: (**A**) Schematic representation of murine *Fas* and *FasL* promoter regions, indicating the localization of consensus E2F motifs detected with Consite at a 0.85 threshold level. The nucleotide positions are numbered relative to the transcriptional start site. Arrows depict the location of primers used for qPCR analysis of immunoprecipitated chromatin sequences, and letters indicate the DNA region analyzed in the qPCRs. (**B**) ChIP-qPCR analyses of *Fas* and *FasL* promoter regions in freshly purified resting (upper panel) and TCR-activated (lower panel) WT lymphocytes. Data from a representative experiment out of three independent experiments (results obtained in Exp. 2 and Exp. 3 are in Supplementary Figure S4). ChIP assays were performed using anti-E2F1, anti-E2F2, and anti-SV40T (irrelevant control) antibodies, and qPCR was performed using primers specific for the *Fas* and *FasL* promoter regions. Analyses of *Rbl1* promoter, a known E2F-target carrying consensus E2F binding sites, and *β-actin* promoter regions were carried out as positive and negative controls, respectively. Data are presented as percentage fold over of input chromatin. The values represent the mean ± SD of qPCR technical triplicates. (**C**) Promoter construct of human *FAS* (-678 to +51), indicating the localization of consensus E2F motifs detected with Consite at a 0.85 threshold level. The nucleotide positions of the promoter are numbered relative to the transcriptional start site. (**D**) Activation of *FAS* promoter-driven firefly luciferase reporter construct after silencing of E2Fs. HCT116 cells were transfected with non-target control siRNA (siCtrl) or with siRNAs specific for *E2F1* (siE2F1), *E2F2* (siE2F2), or their combination (siE2F1 + siE2F2). After 3 h, cells were transfected with FAS-pGL2 along with pRL-TK. Then, 48 h after siRNA transfection, FAS promoter activity was measured. Values are represented as fold-change (mean ± SD) of Relative Luciferase Levels (firefly/*Renilla* = RLU) over siRNA control. n = 3, ** *p* < 0.0001, * *p* < 0.01.

In hematopoietic precursor cells, E2f1 has been shown to repress *Fas* promoter [19]. Furthermore, bioinformatics analysis revealed the existence of E2f consensus motifs in mouse *Fas* and *FasL* promoters (Figure 5A). Thus, as a potential p53-independent mechanism governing Fas and FasL gene expression in T lymphocytes, we speculated that E2f2 could directly repress their transcription.

To test this possibility, we examined the binding of E2f1 and E2f2 to murine *Fas* and *FasL* promoters in resting and TCR-stimulated T cells by Chromatin Immunoprecipitation (ChIP)-qPCR (Figure 5B and Supplementary Figure S4). As a positive control for E2f binding, we amplified a promoter region of *Rbl1*, known to be occupied by E2Fs in quiescent and proliferating cells [21]. As negative controls, we amplified the *β-actin* promoter, which lacks E2F sites, and immunoprecipitated chromatin with an irrelevant anti-SV40 antibody [22]. To evaluate binding to *Fas* promoter, we selected two regions for qPCR analysis: region A corresponds to a distal sequence with previously reported E2f1 binding activity [19]; region B lies near the transcriptional start site, where E2F factors commonly bind to their target genes [23]. This proximal region harbors a consensus E2F-binding motif, but it has not yet been reported to recruit E2F factors. In naïve T cells, we found that E2f1 occupied specifically the region A (Figure 5B), while E2f2 was bound to both regions, although more strongly to the proximal B region (Figure 5B upper panel and Supplementary Figure S4A,B upper panels). Regarding E2f binding to *FasL* promoter, we also examined two regions by qPCR: region A, nearby an E2F motif, and region B, close to the transcriptional start site. In naïve T cells, E2f1 and E2f2 were able to bind both regions. These results suggest that *Fas* and *FasL* are transcriptional targets of E2f factors; although, E2f1 and E2f2 appear to have different binding specificities. Strikingly, E2f binding to *Fas* and *FasL* promoters disappeared in activated lymphocytes (Figure 5B lower panel and Supplementary Figure S4A,B lower panels) coincident with their upregulation, suggesting a repressive activity of E2f2 on these promoters.

To functionally test this possibility, we performed luciferase reporter assays in HCT116 human carcinoma cells, an established model to study Fas/FasL pathway [24,25,26]. We generated a construct containing the regulatory region of human *FAS* promoter, encompassing two E2F consensus motifs (Figure 5C), upstream of the firefly luciferase cDNA. Cells were transfected with this construct after silencing E2F1 and E2F2 individually or in combination (Supplementary Figure S5). Silencing of E2F2, or more potently, combined silencing of E2F1 and E2F2 increased *FAS* promoter activity (Figure 5D). These results are consistent with a role for E2f2 as a transcriptional repressor of *Fas* expression.

### 2.5. E2F2 Loss Enhances Sensitivity to ConA-Induced T-Cell Activation and Immune-Mediated Liver Injury In Vivo

**Figure 6:**
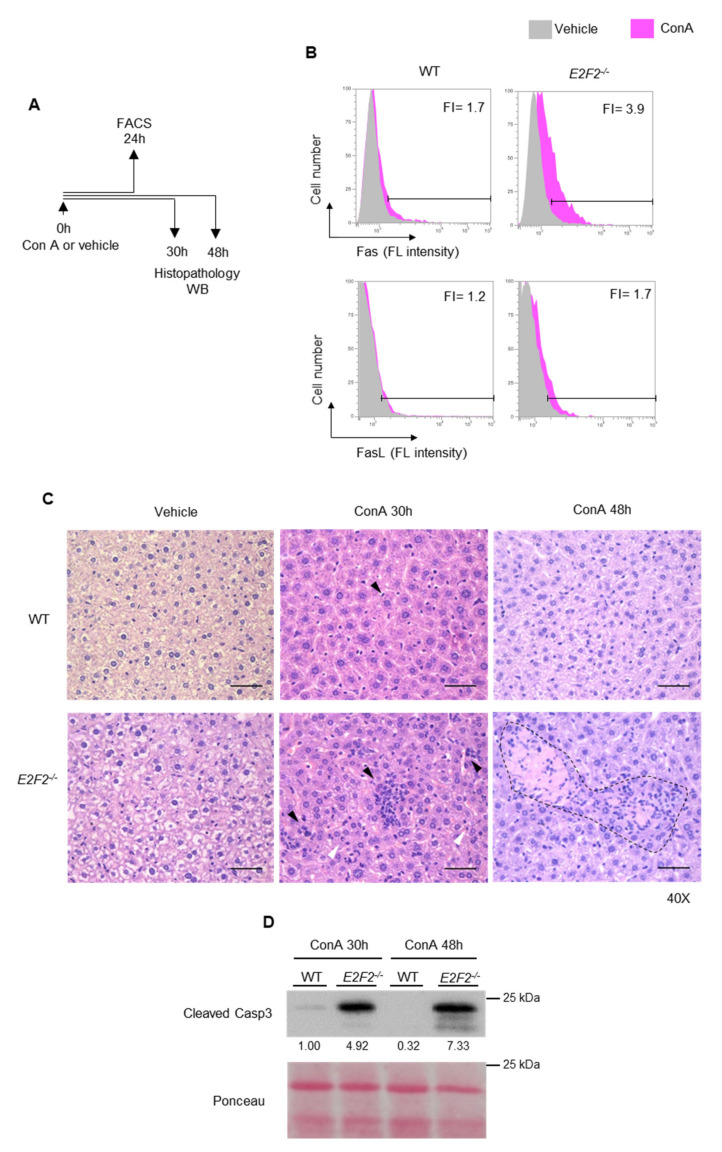
E2F2 loss enhances sensitivity to ConA-induced T-cell activation and immune-mediated liver injury in vivo. (**A**) Experimental set up for FACS analysis, Western blot, and histopathology after the iv injection with ConA (20 mg/kg). (**B**) Results of FACS analysis to detect surface Fas and FasL expression in cells purified from lymph nodes form WT and *E2f2*^−/−^ mice 24 h after injection with ConA (pink histogram) or vehicle control (gray histogram). *X* axis contains the values of fluorescence intensity (FL intensity). The results are expressed as fold increase (FI) of fluorescence intensity mean in ConA-treated samples over vehicle-treated samples. (**C**) Liver sections obtained from WT and *E2f2*^−/−^ animals 30 and 48 h after injection with ConA or vehicle control, and stained with hematoxylin/eosin (H/E, ×40, scale bar = 50 μm). Black arrowheads indicate infiltrated inflammatory cells and white arrowheads point to shrinking nuclei of hepatocytes. Note the enhanced infiltration and enhanced necrotic area (marked with dash line) in *E2f2*^−/−^ animals after the treatment with ConA for 30 h and 48 h, respectively. (**D**) Western blot analysis of Cleaved Caspase 3 in extracts prepared from livers of WT and *E2f2*^−/−^ mice 30 and 48 h after injection with ConA. Ponceau staining was used as loading control. Numbers below the bands correspond to the relative densitometric levels expressed as fold-over WT ConA at 30 h.

To assess the in vivo impact of Fas/FasL activation in *E2f2*^−/−^ mice, animals were treated with concanavalin A (ConA), as shown in Figure 6A, a widely-used model for hepatitis in mice, with important similarities to immune-mediated hepatitis in humans [27]. ConA-induced injury is primarily driven by the activation and recruitment of T cells to the liver, and is dependent on Fas and FasL [28]. Intravenous (iv) injection of ConA led to T cell activation, as shown by an increased percentage of CD69-positive lymphocytes derived from lymph nodes and spleen, compared to mice treated with vehicle (Supplementary Figure S6A,B). Notably, expression of the activation marker CD69 was significantly higher in *E2f2*^−/−^ mice, consistent with a hyperactivated state of these cells. Importantly, the fold increase in surface expression of Fas and FasL after ConA administration was higher in T lymphocytes derived from *E2f2^−/−^* mice compared to WT controls (Figure 6B and Supplementary Figure S6B).

We next examined the liver, as this is the target organ for ConA-mediated lesions [11,27,28]. Inflammatory infiltration was observed sporadically in liver sections obtained from WT mice treated with ConA for 30 h, whereas inflammatory cell accumulation was robust in the livers of *E2f2*^−/−^ treated animals (Figure 6C and Supplementary Figure S6C). Scattered hepatocytes with shrinking nuclei were detected in the livers of treated knockout mice, suggestive of apoptosis. Moreover, tissue architecture was greatly disorganized in the liver of *E2f2*^−/−^ mice treated with ConA for 48 h, showing confluent hepatocyte necrosis (Figure 6C and Supplementary Figure S6C). Western blot analysis performed with protein extracts obtained from livers of the same mice confirmed enhanced cleavage of Casp3 in samples of *E2f2*^−/−^ mice treated with ConA compared to WTs (Figure 6D). These results demonstrate that loss of E2f2 sensitizes mice to Fas/FasL-mediated apoptosis and liver injury.

## 3. Discussion

The mechanisms underlying T-cell homeostasis are still poorly understood. Here we show that E2f2 provides genomic stability to activated T lymphocytes and plays a specific anti-apoptotic function, both in vivo and in vitro. Loss of these activities could underlie the late-onset immune-mediated injury previously observed in *E2f2*^−/−^ mice [4]. Interestingly, the phenotype of *E2f2*-null lymphocytes differs from what is reported for *E2f1*-null lymphocytes. Upon E2f1 loss, the expression of Fas remains unchanged, and apoptosis of activated *E2f1*^−/−^ T cells incubated with agonist anti-Fas is lower than in WTs [20], in contrast to our in vivo findings with *E2f2^−^*^/−^ cells. Our results underscore the differential specificity of E2f1 and E2f2, and introduce T-lymphocyte activation as a suitable experimental model in which to study the mechanisms underlying this specificity.

Based on our findings with *E2f1/E2f2* DKO T cells, we can rule out a role for E2f1, the main proapoptotic E2f member, in the higher apoptotic levels of E2f2-deficient T cells. However, we cannot exclude the possibility that other E2f family members or other factors, such as Myc, known to be expressed in activated lymphocytes [29], may play a role in the observed phenotype. Further work should assess their contribution to apoptosis in our system. Nevertheless, the strong binding of E2f2 to *Fas* and *FasL* promoters suggests that E2f2 is a critical contributor to the direct regulation Fas/FasL-mediated apoptosis.

Expression of E2F2 inhibits apoptosis in other cell systems, such as human cardiomyocytes [30], where it downregulates various apoptosis-related genes, including p53; however, the pathways involved have not been fully elucidated. In melanoma, E2F2 inhibits p53-dependent apoptosis by activating SIRT1 [31], known to deacetylate and inactivate p53 [32]. Sirt1 expression is unlikely to be altered in *E2f2*-null lymphocytes based on our previous negative findings with *E2f2* and *E2f1/E2f2* knockout mice [6,33]. Instead, an increased replication stress that might emanate from the unscheduled S-phase entry in *E2f2*^−/−^ T cells [6] is a possible source of the elevated p53 levels in these cells. Surprisingly, however, p53 only partially accounts for the increased death of *E2f2* knockout cells, as demonstrated by our analyses of *p53/E2f2* DKO cells. Thus, our data reveal the existence of a p53-independent apoptotic mechanism that operates in activated *E2f2*^−/−^ T cells.

**Figure 7:**
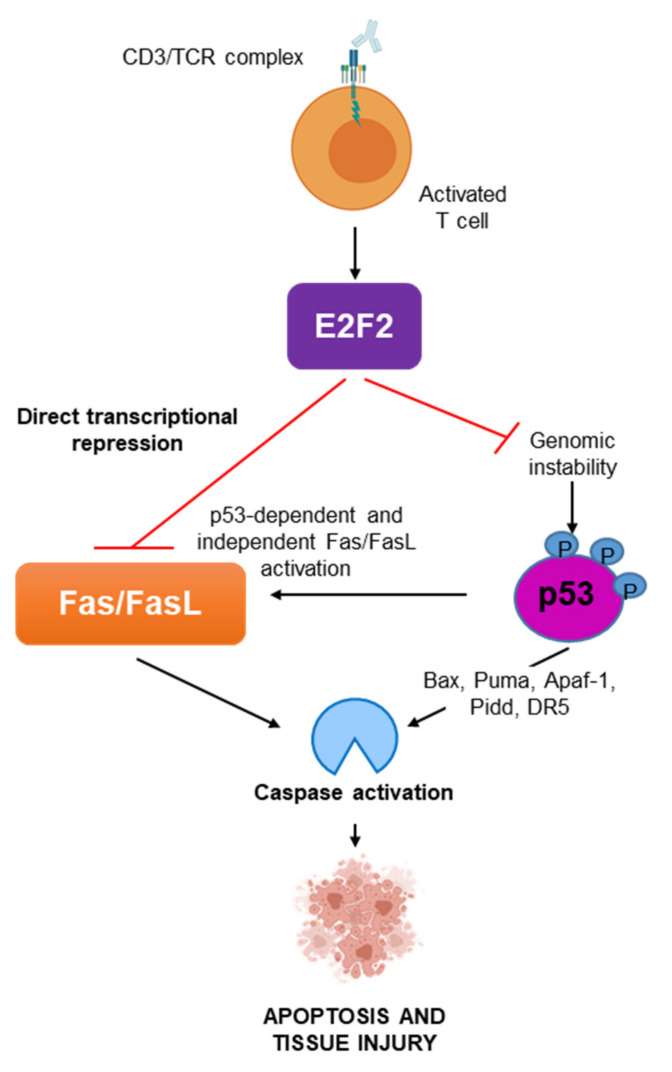
Schematic representation of the proposed mechanism. E2F2 plays an antiapoptotic function in vivo and in vitro. E2F2 prevents p53-dependent Fas/FasL pathway activation and directly represses Fas and FasL expression in activated T-cells to preserve immune cell homeostasis and protect from immune-mediated tissue injury.

Our results indicate that E2f2 negatively regulates *Fas*/*FasL* expression in a p53-independent manner (Figure 7). It has previously been shown that E2f1 represses *Fas/FasL* transcription in murine hematopoietic progenitor cells lacking D-type cyclins [19]; although, this regulation has not been detected in T lymphocytes [20]. We now show recruitment of both E2f1 and E2f2 to the distal promoter region of *Fas* in quiescent T cells, where E2f1 binding has been previously reported [19]. Remarkably, E2f2, but not E2f1, is recruited strongly to the proximal *Fas* promoter region encompassing E2F motifs. Differential recognition of E2F binding-sites has been proposed as a mechanism for transcriptional control specificity among E2F family members [34,35,36]. In line with this, our results suggest that the proximal binding site is critical for the distinct repressive role of E2f2 over *Fas* receptor gene in T lymphocytes. Furthermore, we identify previously unreported E2f2 binding activity to regions in *FasL* promoter harboring E2F motifs. Taken together, our results provide evidence for a novel role for E2f2 as direct repressor of *Fas/FasL* expression in quiescent T cells. Reinforcing this view, knockdown of E2F2 in a different experimental setting (a cancer-derived human cell line) leads to *FAS* promoter activation and transcription. Interestingly, binding of E2fs to *Fas* and *FasL* promoters is drastically diminished after the activation of normal T cells, coinciding with an increase in Fas and FasL expression. This effect is specific to *Fas/FasL* promoters, since typical E2f targets, such as *Rbl1,* remain occupied by E2f1/E2f2 upon activation to trigger their transcription [21]. The putative implication of co-repressors and chromatin modifying factors in E2f2-mediated regulation of *Fas* and *FasL* promoters awaits to be addressed.

Our finding that E2f2 directly represses *Fas* and *FasL* expression has potential clinical implications because several disorders associated to immune-mediated tissue degeneration are linked to hyperactivation of the Fas/FasL pathway [9,10,11,12,13]. Notably, we provide evidence that in vivo activation of T cells leads to robust liver damage in *E2f2*-null mice, likely as a consequence of the aberrant hyper-activation of Fas/FasL, thus reinforcing the clinical relevance of the E2f2–Fas/FasL axis. It would be interesting to confirm these findings in a *Fas*-null context. Based on our data, breeding *E2f2^−/−^* mice with loss-of-function models for the Fas/FasL pathway [7] should prevent ConA-induced liver injury. In immune-mediated injury, activated lymphocytes bearing surface FasL infiltrate non-immune tissue, to elicit a harmful cytolytic activity on target cells expressing Fas. Considering the mechanism proposed in this work (Figure 7) immune-mediated tissue destruction could be abrogated by modulating both lymphoid FasL and non-lymphoid Fas expression thought enhanced E2F2 activity. Our results may also be particularly relevant in hematological malignancies. It is known that a subset of cancers of lymphoid origin exhibit defective Fas/FasL-mediated apoptosis, rendering them resistant to chemotherapy. This is the case of T-cell lymphoblastic lymphoma, cutaneous T-cell lymphoma, or mantle cell lymphoma [37,38,39]. Studies analyzing expression and functional impact of E2F2 in these tumor types are underway, which will provide insight into the feasibility of targeting E2F2 to sensitize cancer cells to Fas/FasL-mediated apoptosis.

## 4. Materials and Methods

### 4.1. Mouse Strains and In Vivo Experiments

Colonies of *E2f2*^−/−^, *E2f2/E2f1*^−/−^, and *p53*^−/−^ mice have been described [4,40,41]. *E2f2*^−/−^ mice were bred to *p53*^−/−^ mice to generate double knockout *E2f2/p53*^−/−^ mice. Colonies are in the C57Bl6:129Sv background. To determine the survival rate after immune-mediated injury, 8-week-old female *E2f2*^−/−^ and WT mice were iv injected with 20 mg/kg of body weight ConA (Cat. C5275, Sigma, St. Louis, MO, USA) [42] and 24 h later tissues were collected and prepared as described previously [33].

### 4.2. Harvest, Purification, Activation, and Treatment of T Lymphocytes

T lymphocytes were harvested from lymph nodes of 6–8-week-old mice and purified as previously described [6]. RPMI supplemented with 10% FCS was used for cell preparation and culture. To study TCR-mediated responses, purified T lymphocytes were stimulated with plate-immobilized antibodies against CD3e (Cat. 553057, BD Bioscience, Franklin Lakes, NJ, USA) at a concentration of 1.5 µg/mL. Where indicated, cells were treated with anti-FasL antibody (5 μg/mL, Cat. 555021, BD Bioscience, Franklin Lakes, NJ, USA).

### 4.3. Analysis of Cell Proliferation, Apoptosis, and Cell Surface Expression

Metabolic activity was assayed using the MTT solution-based kit (Cat. 11465007001, Roche, Basel, Switzerland) as per manufacturer’s suggested protocol. The absorbance was measured at 570 nm. Apoptosis was measured with the Annexin V-FITC Apoptosis Detection Kit I (Cat. 556547, BD Bioscience, Franklin Lakes, NJ, USA) and analyzed by flow cytometry using an Attune-NxT (Thermo Fisher Scientific, Waltham, MA, USA) flow cytometer. For cell surface staining, anti-CD69-FITC (Cat. 553236), anti-Fas-FITC (Cat. 554257), and anti-FasL-PE (Cat. 555293) (BD Bioscience, Franklin Lakes, NJ, USA) antibodies were used.

### 4.4. FAS-Luc Reporter Plasmid Construction

To construct the *FAS*-luc reporter plasmid, a 718 bp fragment (−668 to +51) of the human *FAS* promoter region was amplified by PCR using human genomic DNA as template (See Supplementary Table S1 for primer sequence information). The PCR product was digested with KpnI and HindIII, and inserted into the pGL2-basic luciferase reporter vector (Promega, Madison, WI, USA).

### 4.5. Cell Line Transfection, siRNA-Mediated Knockdown, and Luciferase Activity Assays

Human HCT116 cell line was maintained in DMEM supplemented with 10% FBS. To silence endogenous expression of *E2F1* and *E2F2*, cells were transfected with 6.5 nM of siRNA for *E2F1* (s4405) and *E2F2* (s4409) (Thermo Fisher Scientific, Foster City, CA, USA) using Lipofectamine RNAiMAX (Thermo Fisher Scientific, Foster City, CA, USA).

For luciferase experiments, cells were transfected with 250 ng of *FAS*-pGL2 and 25 ng of the *Renilla* (pRL-TK) plasmids using X-tremeGENE™ HP (Roche, Basel, Switzerland). Using the Dual-Luciferase Reporter Assay System (Promega, Madison, WI, USA), the *FAS*-pGL2 activity was measured 48 h after transfection. Data were normalized to the transfection efficiency estimated by the activity of *Renilla* luciferase, obtaining Relative Luciferase Units (RLU).

### 4.6. Quantitative RT-PCR Analysis

Total RNA was isolated using NZY Total RNA Isolation kit (Nzytech, Lisbon, Portugal). cDNA was synthesized from 1 μg of RNA using High-Capacity cDNA Reverse Transcription Kit (Thermo Fisher, Foster City, CA, USA), and all samples were diluted to the same final cDNA concentration. Real-time PCR was performed on several cDNA dilutions plus 1× SYBR green PCR Master Mix (Thermo Fisher Scientific, Foster City, CA, USA) and 300 to 900 nM of primers for the analyzed genes (sequences in Supplementary Tables S2 and S3). Reactions were carried out in triplicate using a QuantStudio 3 (Thermo Fisher Scientific, Foster City, CA, USA) thermocycler for 40 cycles (95 °C for 15 s and 60 °C for 1 min) after an initial 10 min incubation at 95 °C. Relative amounts of cDNA were normalized to the internal control *Eef1a1* in murine T-cells and *VPS29* in HCT116 cells. Results were expressed as fold-over WT at 0 h.

### 4.7. Protein Extraction and Western Blot Analysis

Cellular extracts were prepared as previously described [6]. Protein concentrations in supernatants were determined using CD Protein Assay (Bio-Rad Laboratories, Hercules, CA, USA). We performed Pounceau staining to confirm correct protein transfer and used it as loading control where indicated. Western blots were performed with 20 μg of total protein extract, using antibodies against Cleaved Caspase 3 (9664S), phospho-Chk1 (Ser345, 133D3) (Cell Signaling, Danvers, MA, USA), phospho-Rpa2 (Ser4/Ser8, A300-245A-M, Thermo Fisher Scientific), phospho-histone H2AX (Ser139, 07-164, Millipore, Billerica, MA, USA), β-actin (A5441) (Sigma), E2F1 (sc-251), Parp-1 (sc-53643), Fas (sc-1023), FasL (sc-6237), Mcm2 (sc-9839), Cyclin A2 (sc-596), Chk1 (sc-7898), p53 (sc-126), and Hsp 90α/β (sc-13119) (Santa Cruz Biotechnologies, Santa Cruz, CA, USA). Immunocomplexes were visualized with horseradish peroxidase-conjugated anti-mouse or anti-rabbit IgG antibodies (Amersham, GE Healthcare Bio-Sciences, Pittsburgh, PA, USA), followed by chemiluminescence detection (ECL, Amersham, GE Healthcare Bio-Sciences, Pittsburgh, PA, USA) with a ChemiDoc camera (Bio-Rad Laboratories, Hercules, CA, USA). Densitometry-based quantification was performed using Fiji software. Relative optical density was calculated by dividing the densitometry of the protein of interest with the respective loading control.

### 4.8. Chromatin Immunoprecipitation (ChIP)

Chromatin immunoprecipitations and the quantification of immunoprecipitate-enriched DNA sequences by real-time PCR were performed as described previously [43]. Sequences of PCR primers are listed in Supplementary Table S4. Antibodies used were E2F1 (sc-251), E2F2 (sc-633), and SV40Tag (sc-147), all from Santa Cruz Biotechnologies (Santa Cruz, CA, USA).

### 4.9. Statistical Analysis

GraphPad Prism 8.0 (GraphPad Software, San Diego, CA, USA) was used for statistical analysis and data representation. Data are given as mean ± SD. Statistical analysis was performed using ANOVA and Fisher’s test. Significance was defined by *p* < 0.05.

## Data Availability

The datasets supporting the conclusions of this article are included within the article (and Supplementary Materials).

## Supplementary Materials

The following supporting information can be downloaded at: https://www.mdpi.com/article/10.3390/ijms23010311/s1.

## References

[1] Hwang J.-R., Byeon Y., Kim D., Park S.-G. (2020). Recent insights of T cell receptor-mediated signaling pathways for T cell activation and development. Exp. Mol. Med..

[2] Chang J.T., Wherry E.J., Goldrath A.W. (2014). Molecular regulation of effector and memory T cell differentiation. Nat. Immunol..

[3] Krammer P.H., Arnold R., Lavrik I.N. (2007). Life and death in peripheral T cells. Nat. Rev. Immunol..

[4] Murga M., Fernandez-Capetillo O., Field S.J., Moreno B., Borlado L.R., Fujiwara Y., Balomenos D., Vicario A., Carrera A., Orkin S.H. (2001). Mutation of E2F2 in Mice Causes Enhanced T Lymphocyte Proliferation, Leading to the Development of Autoimmunity. Immunity.

[5] Zhu J.W., Field S.J., Gore L., Thompson M., Yang H., Fujiwara Y., Cardiff R.D., Greenberg M., Orkin S.H., DeGregori J. (2001). E2F1 and E2F2 Determine Thresholds for Antigen-Induced T-Cell Proliferation and Suppress Tumorigenesis. Mol. Cell. Biol..

[6] Infante A., Laresgoiti U., Fernández-Rueda J., Fullaondo A., Galán J., Díaz-Uriarte R., Malumbres M., Field S.J., Zubiaga A.M. (2008). E2F2 represses cell cycle regulators to maintain quiescence. Cell Cycle.

[7] Nagata S., Golstein P. (1995). The Fas Death Factor. Science.

[8] Levoin N., Jean M., Legembre P. (2020). CD95 Structure, Aggregation and Cell Signaling. Front. Cell Dev. Biol..

[9] Singh N., Cohen P.L. (2012). The T cell in Sjogren’s syndrome: Force majeure, not spectateur. J. Autoimmun..

[10] Pearl-Yafe M., Kaminitz A., Yolcu E., Yaniv I., Stein J., Askenasy N. (2007). Pancreatic Islets Under Attack: Cellular and Molecular Effectors. Curr. Pharm. Des..

[11] Malhi H., Gores G.J. (2008). Cellular and Molecular Mechanisms of Liver Injury. Gastroenterology.

[12] Cai W.-J., Devaux B., Schaper W., Schaper J. (1997). The role of Fas/APO 1 and apoptosis in the development of human atherosclerotic lesions. Atherosclerosis.

[13] Martinez O.M., Krams S.M. (1999). Involvement of Fas-Fas Ligand Interactions in Graft Rejection. Int. Rev. Immunol..

[14] Li X.R., Chong A.S.-F., Wu J., Roebuck K.A., Kumar A., Parrillo J.E., Rapp U.R., Kimberly R.P., Williams J.W., Xu X. (1999). Transcriptional Regulation of Fas Gene Expression by GA-binding Protein and AP-1 in T Cell Antigen Receptor·CD3 Complex-stimulated T Cells. J. Biol. Chem..

[15] Kavurma M.M., Khachigian L.M. (2003). Signaling and transcriptional control of Fas ligand gene expression. Cell Death Differ..

[16] Vousden K.H. (2000). p53: Death Star. Cell.

[17] Müller M., Strand S., Hug H., Heinemann E.M., Walczak H., Hofmann W.J., Stremmel W., Krammer P.H., Galle P.R. (1997). Drug-induced apoptosis in hepatoma cells is mediated by the CD95 (APO-1/Fas) receptor/ligand system and involves activation of wild-type p53. J. Clin. Investig..

[18] Bennett M., Macdonald K., Chan S.-W., Luzio J.P., Simari R., Weissberg P. (1998). Cell Surface Trafficking of Fas: A Rapid Mechanism of p53-Mediated Apoptosis. Science.

[19] Choi Y.J., Saez B., Anders L., Hydbring P., Stefano J., Bacon N.A., Cook C., Kalaszczynska I., Signoretti S., Young R.A. (2014). D-Cyclins Repress Apoptosis in Hematopoietic Cells by Controlling Death Receptor Fas and Its Ligand FasL. Dev. Cell.

[20] Cao Q., Xia Y., Azadniv M., Crispe I.N. (2004). The E2F-1 transcription factor promotes caspase-8 and bid expression, and enhances Fas signaling in T cells. J. Immunol..

[21] Burkhart D.L., Wirt S.E., Zmoos A.-F., Kareta M.S., Sage J. (2010). Tandem E2F Binding Sites in the Promoter of the p107 Cell Cycle Regulator Control p107 Expression and Its Cellular Functions. PLoS Genet..

[22] Laresgoiti U., Apraiz A., Olea M., Mitxelena J., Osinalde N., Rodriguez J.A., Fullaondo A., Zubiaga A.M. (2013). E2F2 and CREB cooperatively regulate transcriptional activity of cell cycle genes. Nucleic Acids Res..

[23] Rabinovich A., Jin V.X., Rabinovich R., Xu X., Farnham P.J. (2008). E2F in vivo binding specificity: Comparison of consensus versus nonconsensus binding sites. Genome Res..

[24] Lim D.Y., Park J.H.Y. (2009). Induction of p53 contributes to apoptosis of HCT-116 human colon cancer cells induced by the dietary compound fisetin. Am. J. Physiol. Liver Physiol..

[25] Fernandes P., O’Donnell C., Lyons C., Keane J., Regan T., O’Brien S., Fallon P., Brint E., Houston A. (2014). Intestinal Expression of Fas and Fas Ligand Is Upregulated by Bacterial Signaling through TLR4 and TLR5, with Activation of Fas Modulating Intestinal TLR-Mediated Inflammation. J. Immunol..

[26] Iessi E., Zischler L., Etringer A., Bergeret M., Morlé A., Jacquemin G., Morizot A., Shirley S., Lalaoui N., Elífio-Esposito S.L. (2015). Death Receptor-Induced Apoptosis Signalling Regulation by Ezrin Is Cell Type Dependent and Occurs in a DISC-Independent Manner in Colon Cancer Cells. PLoS ONE.

[27] Tiegs G., Hentschel J., Wendel A. (1992). A T cell-dependent experimental liver injury in mice inducible by concanavalin A. J. Clin. Investig..

[28] Seino K., Kayagaki N., Takeda K., Fukao K., Okumura K., Yagita H. (1997). Contribution of Fas ligand to T cell-mediated hepatic injury in mice. Gastroenterology.

[29] Wang R., Dillon C.P., Shi L.Z., Milasta S., Carter R., Finkelstein D., McCormick L.L., Fitzgerald P., Chi H., Munger J. (2011). The Transcription Factor Myc Controls Metabolic Reprogramming upon T Lymphocyte Activation. Immunity.

[30] Ebelt H., Hufnagel N., Neuhaus P., Neuhaus H., Gajawada P., Simm A., Müller-Werdan U., Werdan K., Braun T. (2005). Divergent Siblings. Circ. Res..

[31] Zhao H., Tang W., Chen X., Wang S., Wang X., Xu H., Li L. (2017). The NAMPT/E2F2/SIRT1 axis promotes proliferation and inhibits p53-dependent apoptosis in human melanoma cells. Biochem. Biophys. Res. Commun..

[32] Langley E., Pearson M., Faretta M., Bauer U.M., Frye R.A., Minucci S., Pelicci P.G., Kouzarides T. (2002). Human SIR2 deacetylates p53 and antagonizes PML/p53-induced cellular senescence. EMBO J..

[33] Iglesias-Ara A., Zenarruzabeitia O., Buelta L., Merino J.M., Zubiaga A.M. (2015). E2F1 and E2F2 prevent replicative stress and subsequent p53-dependent organ involution. Cell Death Differ..

[34] Araki K., Nakajima Y., Eto K., Ikeda M.-A. (2003). Distinct recruitment of E2F family members to specific E2F-binding sites mediates activation and repression of the E2F1 promoter. Oncogene.

[35] Croxton R., Ma Y., Cress W.D. (2002). Differences in DNA binding properties between E2F1 and E2F4 specify repression of the Mcl-1 promoter. Oncogene.

[36] Freedman J.A., Chang J.T., Jakoi L., Nevins J.R. (2009). A combinatorial mechanism for determining the specificity of E2F activation and repression. Oncogene.

[37] Villa-Morales M.C., Cobos M.A., González-Gugel E., Alvareziglesias V., Martínez B., Piris M.A., Carracedo A., Benitez J.A., Fernández-Piqueras J. (2014). FAS system deregulation in T-cell lymphoblastic lymphoma. Cell Death Dis..

[38] Wu J., Nihal M., Siddiqui J., Vonderheid E.C., Wood G.S. (2009). Low FAS/CD95 Expression by CTCL Correlates with Reduced Sensitivity to Apoptosis that Can Be Restored by FAS Upregulation. J. Investig. Dermatol..

[39] Hofmann W.-K., De Vos S., Tsukasaki K., Wachsman W., Pinkus G.S., Said J.W., Koeffler H.P. (2001). Altered apoptosis pathways in mantle cell lymphoma detected by oligonucleotide microarray. Blood.

[40] Iglesias A., Murga M., Lasresgoiti U., Skoudy A., Bernales I., Fullaondo A., Moreno B., Lloreta J., Field S.J., Real F.X. (2004). Diabetes and exocrine pancreatic insufficiency in E2F1/E2F2 double-mutant mice. J. Clin. Investig..

[41] Donehower L.A., Harvey M., Slagle B.L., McArthur M.J., Montgomery C.A., Butel J., Bradley A. (1992). Mice deficient for p53 are developmentally normal but susceptible to spontaneous tumours. Nat. Cell Biol..

[42] Shinohara Y., Tsukimoto M. (2018). Adenine Nucleotides Attenuate Murine T Cell Activation Induced by Concanavalin A or T Cell Receptor Stimulation. Front. Pharmacol..

[43] Mitxelena J., Apraiz A., Rodriguez J.V., Malumbres M., Zubiaga A.M. (2016). E2F7 regulates transcription and maturation of multiple microRNAs to restrain cell proliferation. Nucleic Acids Res..

