# Supplementary material for: E2f2 Attenuates Apoptosis of Activated T Lymphocytes and Protects from Immune-Mediated Injury through Repression of Fas and FasL
Source: Int J Mol Sci. 2021 Dec 28;23(1):311. doi: 10.3390/ijms23010311 (PMC8745065; doi:10.3390/ijms23010311)
Supplement: Supplementary file 1 [file ijms-23-00311-s001.zip › Supp_figure_legends.pdf]

SUPPLEMENTARY FIGURE LEGENDS

Suppl. Figure S1. *E2f1* is upregulated in *E2f2*$^{-/-}$ T cells, but it is not responsible for the increased apoptosis of *E2f2*$^{-/-}$ cells. **A)** Representative Western blot analysis of E2F1 in extracts prepared from freshly purified WT and *E2f2*$^{-/-}$ T cells unstimulated or after stimulation with anti-CD3 for the indicated time. Expression of Hsp 90α/β was used as loading control. Similar results were obtained in at least 4 independent experiments. **B)** Annexin V-FITC and PI staining followed by FACS analysis was performed with TCR-stimulated T cells from WT, *E2f2*$^{-/-}$, *E2f1*$^{-/-}$ and *E2f2/ E2f1*$^{-/-}$ mice. Results of early apoptosis are expressed as percentage of cells (mean±SD) from 3 independent experiments.

Suppl. Figure S2. Silencing of *E2F2* results in increased expression of *FAS* in the human cell line HCT116. RT-qPCR analysis of *E2F2* and *FAS* in HCT116 cells transfected with non-target siRNA control (siCtrl) or with siRNA specific for *E2F2* (siE2F2). mRNA expression values were normalized to the expression of *VPS29*, used as standard control. Data are represented as fold-change (mean±SD) relative to siRNA control. n=3, ** $p < 0.0001$, *$p < 0.01$.

Suppl. Figure S3. Elimination of p53 in an E2f2-deficient background completely prevents upregulation of its canonical targets. Reverse transcription Q-PCR analysis of *Puma, Apaf-1, Pidd and Dr5* in WT, *E2f2*$^{-/-}$, *p53*$^{-/-}$ and *E2f2/p53*$^{-/-}$ purified T cells unstimulated or after stimulation with anti-CD3 during the indicated time. *Eef1a1* was used as normalization control. Results are expressed as fold over WT at 0h (mean±SD) from 3 independent experiments. *$p < 0.05$, ns= non-significant.

Suppl. Figure S4. ChIP-qPCR analyses of *Fas* and *FasL* promoter regions in two independent experiments showing similar results to those in Figure 5D. ChIP assays for experiment 2 (**A**) and experiment 3 (**B**) were performed using anti-E2F1, anti-E2F2 and anti-SV40T (irrelevant control) antibodies, and qPCR was performed using primers specific for *Fas* and *FasL* promoter regions, as shown in Figure 5A. Analyses of *Rbl1* promoter and *β-actin* promoter regions were carried out as positive and negative controls, respectively. Data are presented as percentage fold over of input chromatin. The values represent the mean ± SD of qPCR technical triplicates.

Suppl. Figure S5. Efficiency of E2F1 and E2F2 silencing in the experiment shown in figure 5D. RT-qPCR analysis of HCT116 cells transfected with non-target control siRNA (siCtrl) or with siRNAs specific for *E2F1* (siE2F1), *E2F2* (siE2F2) or their combination (siE2F1+ siE2F2). mRNA expression values were normalized to the expression of *VPS29*, used as standard control. Data are represented as fold-change (mean±SD) relative to siRNA control. n=3, **p<0.0001, *p<0.01.

Suppl. Figure S6. Results of FACS analysis to detect surface CD69 expression in cells purified from lymph nodes (**A**) and CD69, Fas and FasL from spleen (**B**) from WT and *E2f2*$^{-/-}$ mice 24h after injection with ConA (pink histogram) or vehicle control (gray histogram). The results are expressed as fold increase (FI) of fluorescence intensity mean (FL mean) in Con A-treated samples over vehicle-treated samples. **C)** Liver sections obtained from WT and *E2f2*$^{-/-}$ animals 30 and 48h after injection with ConA or vehicle control, and stained with hematoxylin/eosin (H/E, x40, scale bar= 50 µm). Black arrowheads indicate infiltrated inflammatory cells and white arrowheads point to shrinking nuclei of hepatocytes. Note the enhanced infiltration and liver damage in *E2f2*$^{-/-}$ animals after the treatment with ConA.
