# Supplementary material for: E2f2 Attenuates Apoptosis of Activated T Lymphocytes and Protects from Immune-Mediated Injury through Repression of Fas and FasL
Source: Int J Mol Sci. 2021 Dec 28;23(1):311. doi: 10.3390/ijms23010311 (PMC8745065; doi:10.3390/ijms23010311)
Supplement: Supplementary file 1 [file ijms-23-00311-s001.zip › Supplementary tables.pdf]

Suppl. Table S1. Nucleotide sequences of primers used for construction of FAS promoter

Primer	Sequence	R.E.* added	Size (bp)
**FAS_F**	TGCAGGGTACCCTAAGGGGCCCTCCCTTTTC	KpnI	718
**FAS_R**	TGCAGGCTAGCAGCTTCCCCAACTCCGTACT	HindIII	

*R.E., restriction endonuclease

Suppl. Table S2. Forward and reverse oligonucleotides used for quantitative RT-PCR in murine cells

Gene	Forward (5´-3´)	Reverse (5´-3´)
Bax	ATCCAGGATCGAGCAGGGA	TCCTCTGCAGCTCCATATTGC
Puma	ACGACCTCAACGCGCAGTA	GTCGGTGTCGATGCTGCTC
Apaf-1	TGCTCAGCGGATAAGAAGGT	TCCCAGAGCTTGAGGAAGAA
Pidd	AGCGTATCCGGCATGAATTC	CGCTCAGCCCAAGAGAAGAG
Dr5	CCCATCAAGAGGACCCTGTCTA	TCTCTGCAAGGCTTGCAGTTC
Fas	GAGGCCCATTTTGCTGTC	CCCCATTCATTTTGCAGTCCT
FasL	GAGTGTGGCCCATTTAACAGG	CAGAGATCAGAGCGGTTCCATA
Eef1a1	CATCGATCGTCGTTCTGGTAAGA	CGACCAAGTGGAGGGTAGTCA

Suppl. Table S3. Forward and reverse oligonucleotides used for quantitative RT-PCR in human cells

Gene	Forward (5´-3´)	Reverse (5´-3´)
E2F1	TGACATCACCAACGTCCTTGA	CTGTGCGAGGTCCTGGGTC
E2F2	ACGTGCTGGAAGGCATCC	GCT CCG TGT TCA TCA GCT CC
FAS	GGGAAGGAGTACACAGACAAAG	GGTCCGGGTGCAGTTTATT
VPS29	CTCTGGCTGGTGATGTTCATA	CTGTCCAACAGTCACAACTTTC

Suppl. Table S4. Forward and reverse oligonucleotides used for quantitative ChIP-PCR

Promoters	Forward (5´-3´)	Reverse (5´-3´)
Fas-A	CAGTCTCTGTGAGGTCATGTG	AAGAGGAGGAGGCAGGAA
Fas-B	CGATTTCTGGGAAGACCTGAATAG	TAGACTGCCTGTGGGTATGT
FasL-A	GGCCAGAAGACTGAAGTCAAA	ACCATCACACAGCACATACTAAA
FasL-B	AGCTGCGGAAGAGCTAATG	GCTAACTGAGAAGGCTGGTAAA
Rbl1	TTAGAGTCCGAGGTCCATCTTCT	GGGCTCGTCCTCGAACATATCC
β-actin	GGGAGTGACTCTCTGTCCATTCA	ACCCTATTTGTGTGGCCTCTTG
