# Supplementary figures and images for: E2f2 Attenuates Apoptosis of Activated T Lymphocytes and Protects from Immune-Mediated Injury through Repression of Fas and FasL
Source: Int J Mol Sci. 2021 Dec 28;23(1):311. doi: 10.3390/ijms23010311 (PMC8745065; doi:10.3390/ijms23010311)

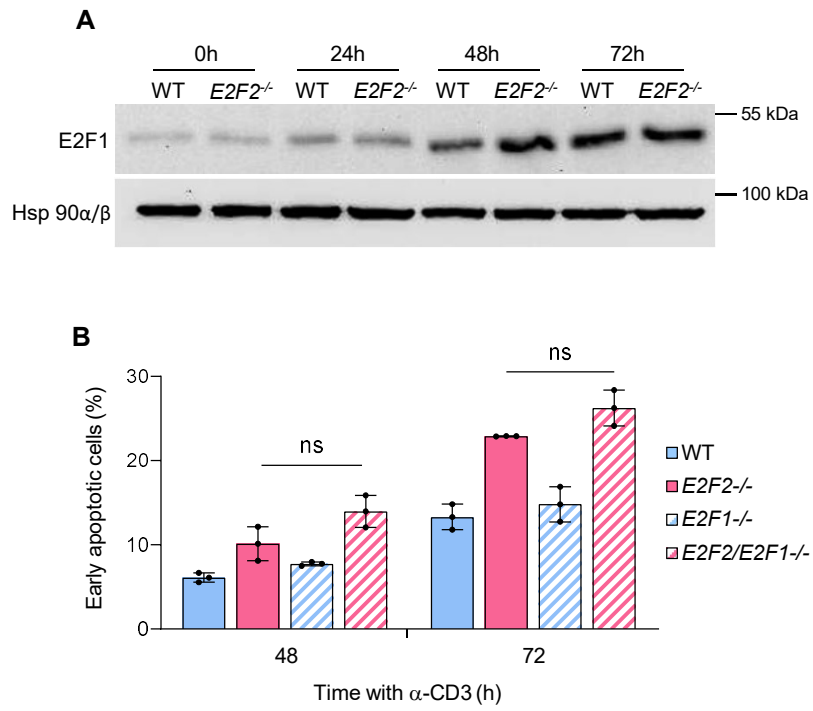

**Suppl. Fig. S1**

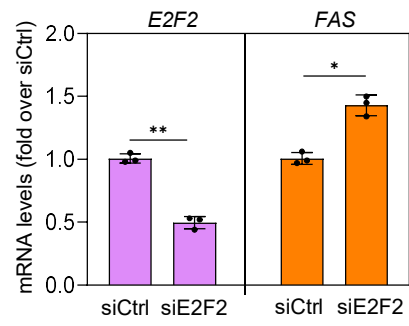

**Suppl. Fig. S2**

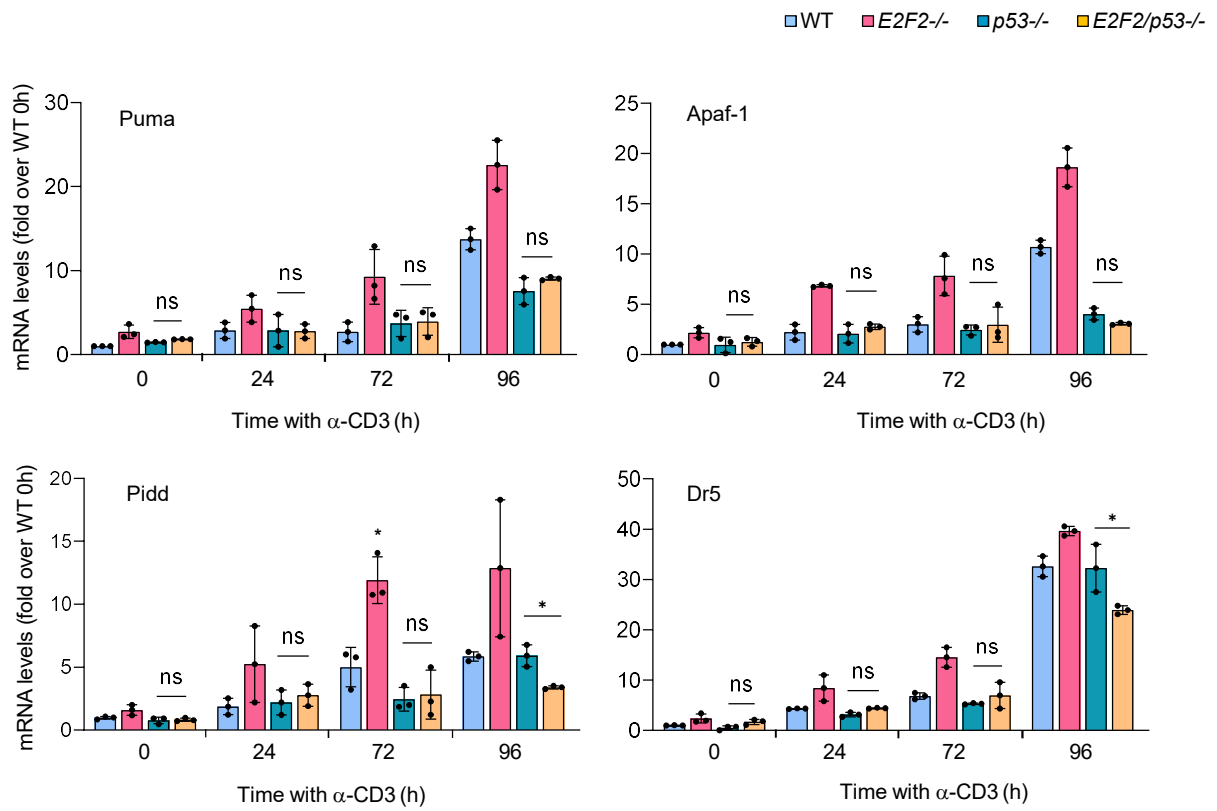

**Suppl. Fig. S3**

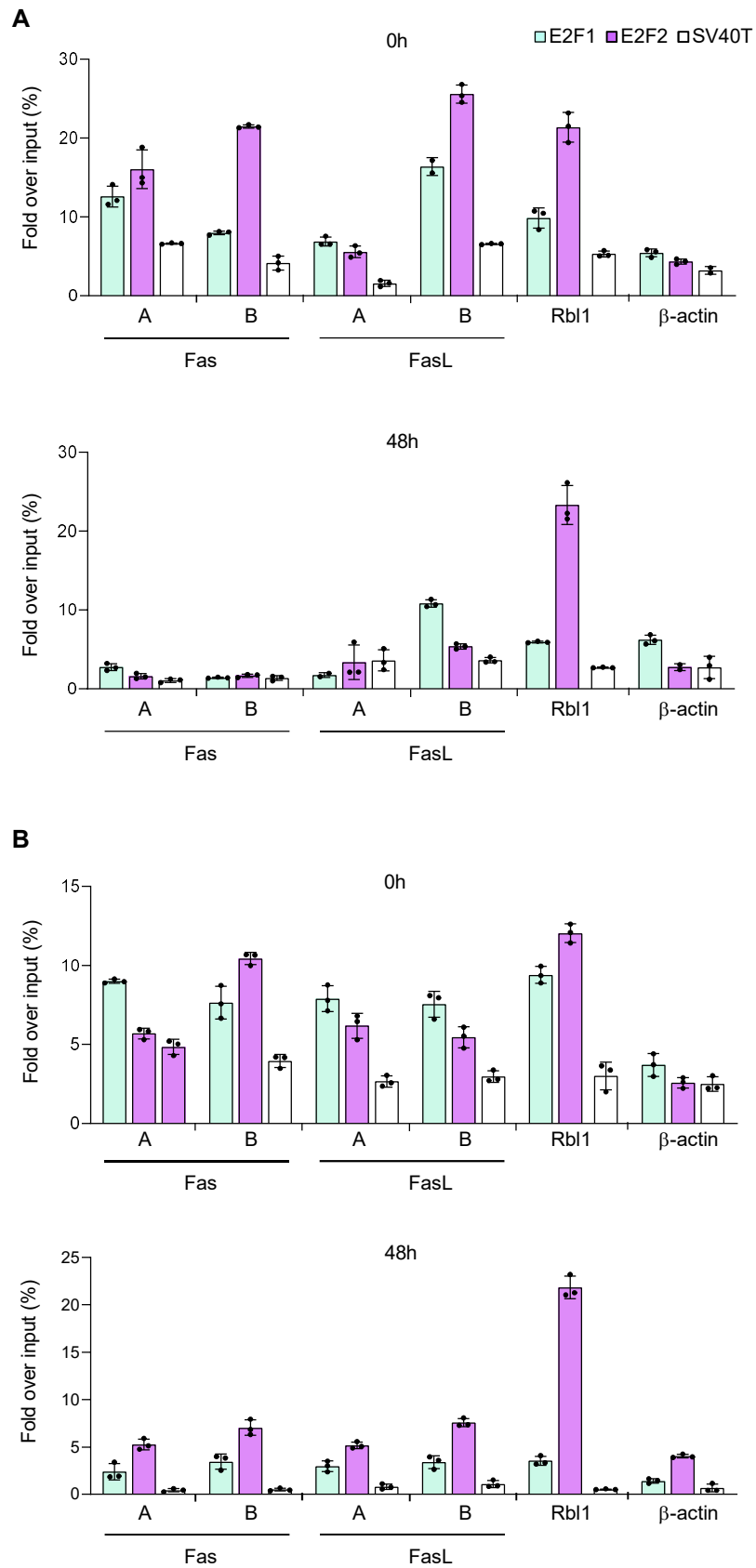

Suppl. Fig. S4

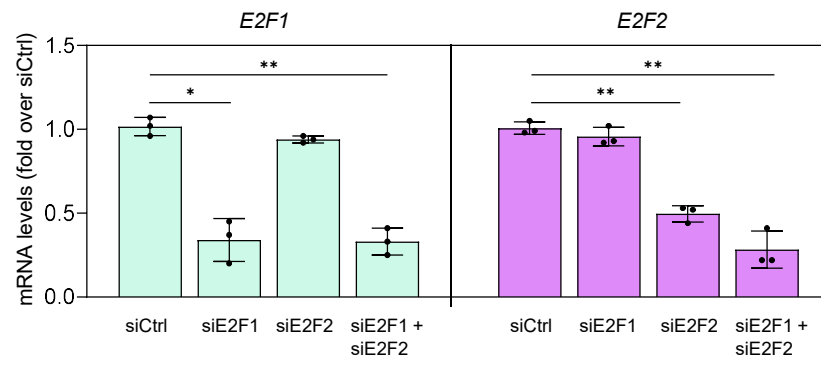

**Suppl. Fig. S5**

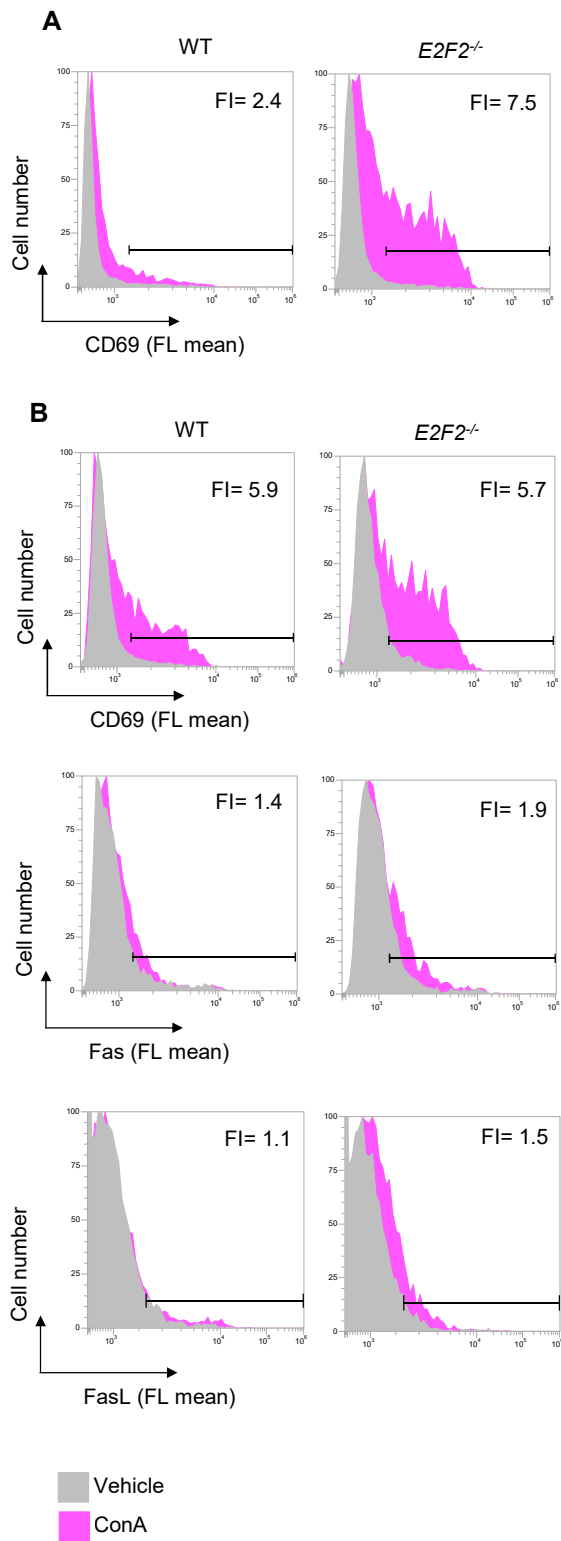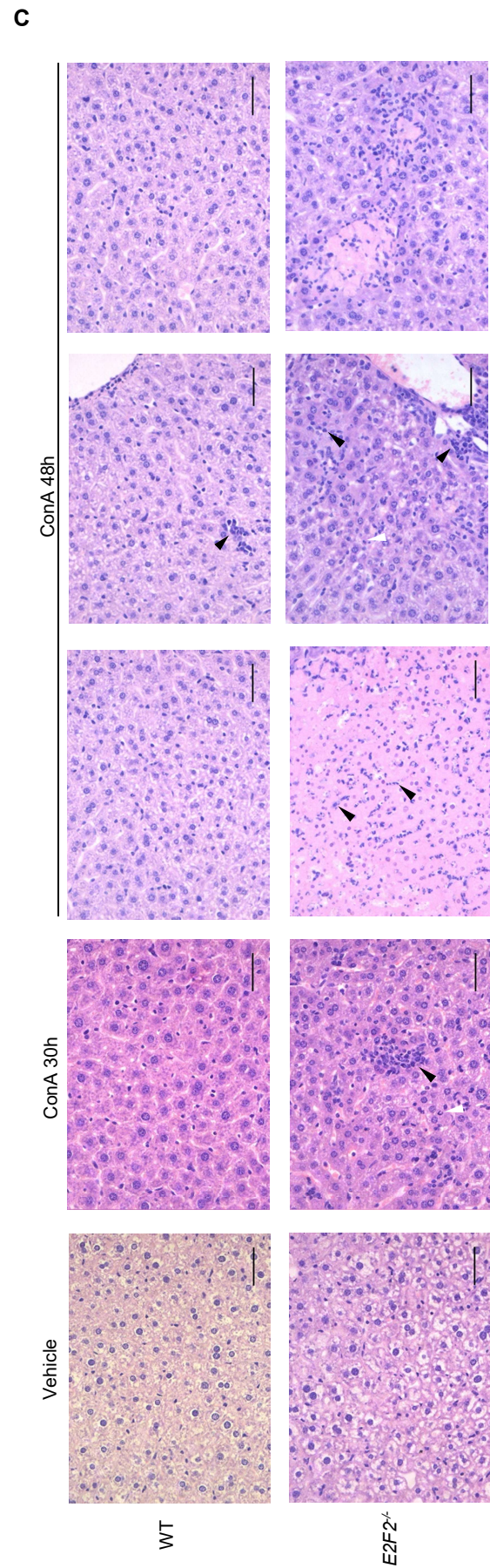

Suppl. Fig. S6

Supplement: Supplementary file 1 [file ijms-23-00311-s001.zip › Supp_figures.pdf]
